# Data on the removal of turbidity from aqueous solutions using polyaluminum chloride

**DOI:** 10.1016/j.dib.2018.08.024

**Published:** 2018-08-15

**Authors:** Sajad Mazloomi, Soudabeh Ghodsei, Paria Amraei, Ziaeddin Bonyadi

**Affiliations:** a Department of Environmental Health Engineering, Ilam University of Medical Sciences, Ilam, Iran; b Department of Environmental Health Engineering, Social Determinants of Health Research Center, Mashhad University of Medical Sciences, Mashhad, Iran

**Keywords:** Polyaluminum chloride, Turbidity, Coagulation, Floculation

## Abstract

Polyaluminum chloride (PAC) is claimed to be superior to conventional coagulants because of higher removal of particulate and/or organic matters as well as inherent advantages of lower alkalinity consumption and lesser sludge production. 1000 mL of the reaction mixture was examined using parameters, including PAC dose (5–10 mg/L), pH (4–9), and turbidity (1.9 NTU). The content was stirred at 120 rpm for 1 min. Thereafter, the turbidity of water samples was measured using a P2100 turbidimeter. Data indicated that the maximum removal efficiency of turbidity (97.74%) obtained under the PAC doses of 4 and 10, and the pH of 8. There is not a significant relationship between the different dosages of PAC (*P*-value > 0.05), but the influence of pH on the removal of turbidity was significant (*P*-value < 0.05). Based on the dataset, the removal efficiency of turbidity was depended on PAC and pH.

**Specifications Table**

**Table:** 

Subject area	Environment
More specific subject area	Water treatment
Type of data	Figure
How data was acquired	Jar tests were carried out in a six-paddled tester (the model of HACH-25632-02) with six 1-L beakers. The turbidity of water samples was measured using a P2100 turbidimeter (HACH).
Data format	Observations, analyzed
Experimental factors	1000 mL of the reaction mixture was examined using parameters, including PAC dose (5–10 mg/L), pH (4-9), and turbidity (1.9 NTU).
Experimental features	Turbidity removal carried out by PAC.
Data source location	Department of Environmental Health, Faculty of Health, Ilam University of Medical Science, Ilam, Iran
Data accessibility	Data are with this article.

**Value of the data**

• Data on the effect of different factors (PAC dose (5–10 mg/L) and pH (4–9)) for turbidity removal covered.
• Data shown here can be useful for other groups working or studying in the field of application of PAC in remediation processes.
• Our data showed that PAC remove turbidity from aqueous solutions.

## 1 Data

**Fig. 1:**
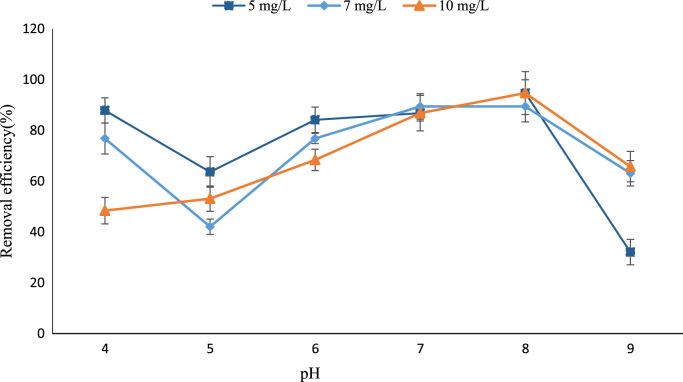
The combined effect of PAC dose and pH on the removal efficiency of turbidity.

PAC, a pre-hydrolyzed Al-based polymer coagulant, has been found to be less sensitive to temperature and thus more suitable for application at lower temperatures (1, 2). It has been considered as a useful experimental method to remove turbidity from aqueous solutions. In this article, the effects of PAC dose and pH on the removal efficiency of turbidity were investigated. Fig. 1 displays the combined effect of PAC dose and pH on the removal efficiency of turbidity. The data indicated that the maximum removal efficiency of turbidity (97.74%) obtained under the PAC doses of 4 and 10, and the pH of 8. There is not a significant relationship between the different dosages of PAC (*P*-value > 0.05), but the effect of pH on the removal of turbidity was significant (*P*-value < 0.05). Fooladvand et al. [1] indicated that increasing of pH value yielded a greater THMFP concentration for Karoon River water (3). Ramavandi and Farjadfard [2] determined that the wastewater could be effectively treated by using a coagulation/flocculation process (4). Ramavandi [3] reported that the FCE removed more than 95.6% of all initial turbidity concentrations (50–300 NTU) (5).

## 2 Experimental design, materials and method

### 2.1 Materials

All chemicals used in the experiments were of reagent grade. All solutions were prepared using distilled water. PAC, HCl and NaOH were provided by Merck company.

### 2.2 Preparation of reaction mixtures

In this work, the use of PAC for the treatment of water entry into the water treatment plant of Ilam was evaluated. Raw water obtained from a reservoir was transferred into each beaker, which was maintained in the thermostat at the temperature of 3–8 °C and kept during the jar test. To determine the optimum dose of PAC, Jar tests were applied in a six-paddled tester (the model of HACH-25632-02) with six 1-L beakers. 1000 mL of the reaction mixture was tested using parameters, including PAC dose (5–10 mg/L), pH (4–9), and turbidity (1.9 NTU). The content was agitated at 120 rpm for 1 min. The mixing speed was then decreased to 30 rpm and was maintained for 20 min, followed by a sedimentation stage for 20 min. The initial pH in the reaction solution was adjusted to a desired value with 1 M NaOH or 1 M HCl.

### 2.3 Analytical methods

After sedimentation, five mL of sample was picked up from approximately 2 cm below the water surface for analysis. The turbidity of water samples was determined using a P2100 turbidimeter (HACH).

Based on Eq. (1), the residual turbidity of sample was RT_S_. The same coagulation test was performed with no coagulant as the blank. The residual turbidity in the blank was RT_B_. Coagulation activity was measured as:

(1) $$\mathrm{Coagulation\backslash activity}(\%)=\frac{{\mathrm{RT}}_{B}-{\mathrm{RT}}_{S}}{{\mathrm{RT}}_{B}}\times 100$$

### 2.4 Statistical analysis

The significance of means within the groups of experimental data was evaluated using one-way analysis of variance (one-way ANOVA).
