# Supplementary material for: Data on the removal of turbidity from aqueous solutions using polyaluminum chloride
Source: Data Brief. 2018 Aug 15;20:371–4. doi: 10.1016/j.dib.2018.08.024 (PMC6117952; doi:10.1016/j.dib.2018.08.024)
Supplement: Supplementary file 1 — Supplementary material [file mmc1.doc]

Conflict of Interest and Authorship Conformation Form

Please check the following as appropriate:

* All authors have participated in (a) conception and design, or analysis and interpretation of the data; (b) drafting the article or revising it critically for important intellectual content; and (c) approval of the final version.

* This manuscript has not been submitted to, nor is under review at, another journal or other publishing venue.

* The authors have no affiliation with any organization with a direct or indirect financial interest in the subject matter discussed in the manuscript
